# Investigation of the effects of different surgical procedures for pelvic organ prolapse on sexual function and partner satisfaction: A retrospective observational study

**DOI:** 10.1097/MD.0000000000048053

**Published:** 2026-03-13

**Authors:** Elif Ucar, Ozan Dogan, Erdem Gürkan, Murat Yassa

**Affiliations:** a Department of Midwifery, Faculty of Health Sciences, Istanbul Esenyurt University, Istanbul, Turkey; b Department of Obstetrics and Gynecology, Faculty of Medicine, Istanbul Nisantasi University, Istanbul, Turkey; c Department of Obstetrics and Gynecology, Kocaeli City Hospital, Kocaeli, Turkey; d Department of Obstetrics and Gynecology, Acibadem University, Acibadem Kartal Hospital, Istanbul, Turkey.

**Keywords:** cervical ring, laparoscopic uterosacral ligament suspension, sacrospinous ligament fixation, sexual functions, uterine prolapse

## Abstract

This study evaluates the impact of different surgical methods on sexual function and partner satisfaction in women with isolated apical pelvic organ prolapse. It compares uterus-sparing procedures (laparoscopic lateral mesh suspension [LLMS] and laparoscopic uterosacral ligament suspension [LUSLS]) with vaginal hysterectomy (VH) and bilateral sacrospinous ligament fixation (SSLF). This retrospective study included 20 patients with isolated apical uterine prolapse between March 2020 and March 2022. Patients were divided into 2 groups: Group 1 underwent LLMS and LUSLS, while Group 2 received VH and SSLF. Preoperative and postoperative sexual function was assessed using the Female Sexual Function Index, Quality of Sexual Experience Scale, Pelvic Organ Prolapse Symptom Score, and Female Genital Self-Image Scale. Partner sexual function was evaluated using the International Erectile Dysfunction Index. Both groups showed significant postoperative improvements in sexual function, with Group 1 demonstrating greater enhancement across all Female Sexual Function Index subdomains. Although vaginal length decreased in both groups, no significant difference was observed between them. Partner erectile function scores were significantly higher in Group 1, which also showed better overall sexual satisfaction. Uterus-sparing surgeries, particularly LLMS and LUSLS, lead to better sexual function, partner satisfaction, and recovery compared to VH with SSLF. Preserving the uterus and cervix may help maintain sexual function and improve patient outcomes, emphasizing the importance of anatomical and functional preservation in pelvic organ prolapse surgery.

## 1. Introduction

The increase in average life expectancy has also led to a rise in the prevalence of pelvic organ prolapse (POP). Although POP can occur in women of all age groups, its prevalence increases with age, peaking among women aged 60 to 69 years.^[1]^ One study estimates that by 2050, the prevalence of POP could rise by up to 46%, potentially affecting approximately 5 million women.^[2]^

POP is defined as the descent of the anterior and posterior vaginal walls, the uterus, or the vaginal apex, either individually or in combination, due to the weakening of the supporting tissues of the pelvic organs.^[3]^ Anterior wall prolapse is 2 to 3 times more common than posterior or apical prolapse, with isolated apical prolapse being the least frequent.^[4]^ Normal pelvic support is provided by the levator ani muscle and connective tissue attachments of the vagina to the pelvic sidewalls. The vagina extends horizontally over the levator ani muscle, but when these structures are damaged, the vaginal opening widens, shifting the primary support to connective tissue attachments. Increased intra-abdominal pressure, as well as variations in bladder and rectal activity and fullness, contribute to the manifestation of POP symptoms.

Pelvic floor muscles, particularly the levator ani and perineal membrane, play a crucial role in female sexual function and can influence sexual satisfaction. These muscles are primarily responsible for involuntary contractions during orgasm.^[5]^ Sexual dysfunction is a common complaint among women with POP, negatively affecting various aspects of sexual function. Consequently, POP is known to adversely impact sexual activity, body image, and overall quality of life.^[6]^ Studies have shown that patients experience pain during intercourse, leading to a decline in quality of life, while severe POP symptoms contribute to poor body image and negatively affect sexual function.^[7]^

The primary goal of POP surgery is to alleviate prolapse-related symptoms; however, preserving sexual function is also a significant concern, particularly for sexually active patients.^[8]^ In recent years, perspectives on sexuality and the psychological significance of reproductive organs have evolved in Western countries, leading to a growing preference for uterus-preserving surgeries.^[9]^ Studies have indicated that women undergoing surgery for POP may be inclined to prefer uterus-preserving procedures.^[10]^ Literature reviews indicate that women, particularly those of reproductive age, tend to opt for uterus-preserving surgeries due to concerns about potential impacts on sexual function.^[11]^

Research suggests that preserving the cervix during colpotomy and maintaining paracervical ligaments enhance apical vaginal support, helping to maintain optimal vaginal length and, consequently, better sexual function.^[12]^ While laparoscopic sacrocolpopexy and sacrohysteropexy are considered the gold standard for apical POP treatment, sacral dissection during these procedures can lead to significant nerve, vascular, and ureteral injuries.^[13]^ Therefore, laparoscopic lateral mesh suspension (LLMS) has emerged as a safe and viable alternative.^[14]^ LLMS preserves the uterus, reduces the risk of complications, and ultimately improves both sexual function and patient satisfaction.^[15]^

In this study, we investigated the impact of surgical procedures both uterus preserving and non-uterus preserving on sexual function in patients with isolated apical prolapse and their partners. Our aim is to elucidate the potential effects of these surgical approaches on sexual life, thereby enabling patients to make more informed decisions when selecting a surgical treatment and ultimately improving patient satisfaction.

We hypothesized that uterus-preserving surgical techniques, such as LLMS and laparoscopic uterosacral ligament suspension (LUSLS), would lead to better postoperative sexual function and higher partner satisfaction compared to vaginal hysterectomy (VH) combined with bilateral sacrospinous ligament fixation (SSLF).

## 2. Materials and methods

This study included 20 patients who presented with POP and were diagnosed with isolated apical uterine prolapse at a private clinic between March 2020 and March 2022. The study was designed retrospectively. Ethical approval was obtained from the Ethics Committee of Istanbul Esenyurt University with the approval number 2024-02 on March 05, 2024.

Prior to data analysis, a post hoc power analysis was conducted using the G*Power 3.1 software (latest version 3.1.9.7; Heinrich-Heine-Universität Düsseldorf, Düsseldorf, Germany). Based on the observed effect sizes in the Female Sexual Function Index (FSFI) and Quality of Sexual Experience Scale (QSES) domains, the achieved power was calculated as 90% with an alpha level of 0.05, indicating adequate statistical power for the detected differences.

Additionally, a sample size calculation revealed that a minimum of 9 patients per group would be required to detect a moderate to large effect size (*d* = 1.2) with 90% power and a 5% significance level in a two-tailed comparison. Therefore, the inclusion of 10 patients in each group meets this threshold and supports the validity of our statistical analysis.

Patient data were retrieved from the hospital’s digital records and patient files. Medical history and gynecological examination notes were recorded. All patients were examined by the same physician using a GE Voluson E8 OB/GYN ultrasound device. Vaginal length measurements were performed during the Valsalva maneuver, referencing point C of the Pelvic Organ Prolapse Quantification System (POP-Q). In the lithotomy position, with the torso at a 45-degree angle and performing maximal Valsalva maneuver, the distance from the hymenal ostium to the Douglas pouch (D point in POP-Q) was recorded as vaginal length. Cervical length was measured in the coronal plane, averaging at least 3 measurements taken between the internal and external os with an empty bladder.

A cervical anatomical length of 5 cm or more was defined as “cervical elongation.”^[16]^ Postoperative apical prolapse, cystocele, and rectocele staging were recorded for all patients using the POP-Q classification system.^[17]^

**Figure 1.**
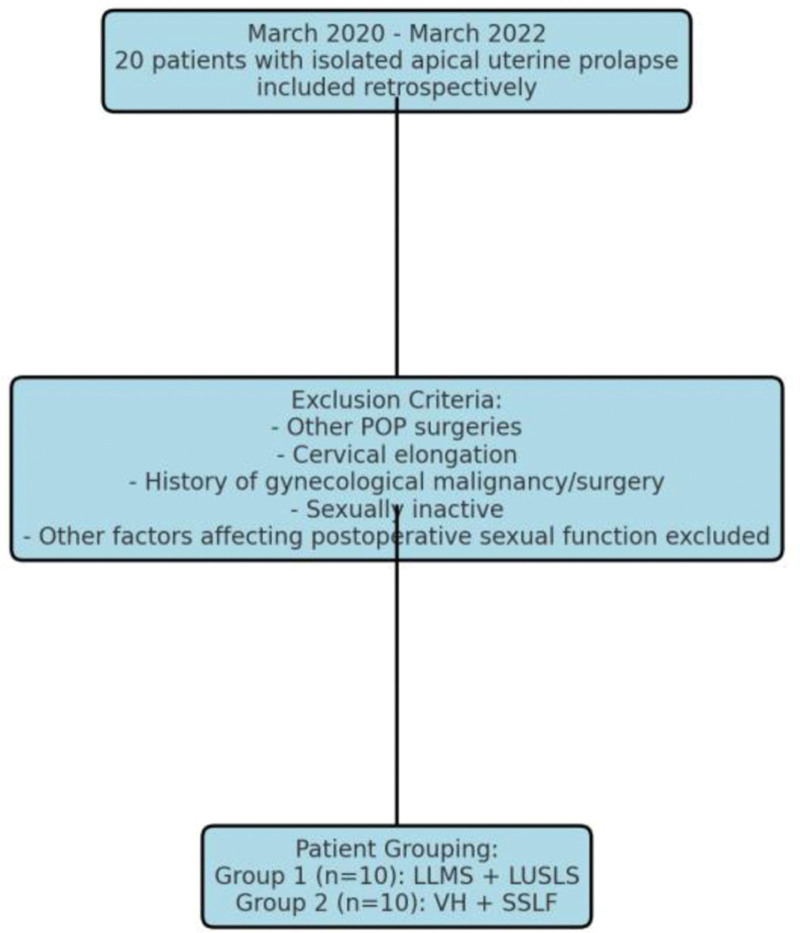
Flowchart: study design overview.

Patients with symptomatic stage 2 or higher apical prolapse who consented to POP surgery were included in the study. All participants were sexually active and had a partner. The following patients were excluded: those with a history of previous POP surgery, previous cystocele and/or rectocele surgery, surgical interventions affecting erogenous zones of the vagina, cervical elongation, previous gynecological surgery, gynecological malignancy or premalignant lesions (or suspected malignancy), patients who had received chemotherapy and/or radiotherapy, those who opted for conservative treatments such as pessary use, and those whose partners had erectile dysfunction (Fig. 1).

The patients were divided into 2 groups. Patients who requested uterus-preserving surgery were allocated to the first group based on patient preference, while the remaining patients were assigned to the comparison surgical group using a predefined allocation strategy aimed at approximating random assignment in a retrospective observational design. Each group consisted of 10 patients. Group 1 underwent LLMS and LUSLS. Group 2 underwent VH with bilateral SSLF. Patients were given detailed information about the procedures, and informed consent was obtained. All surgeries were performed by the same surgeon using standardized surgical techniques (Fig. 1).

For patients who underwent hysterectomy, the anatomical reference point for vaginal length measurement was adjusted, as the POP-Q point C (cervix) is no longer applicable. Therefore, the vaginal cuff was used as a surrogate reference, and the distance from the hymenal opening to the apex of the cuff was measured during maximal Valsalva.

Although this is not the conventional use of point C, this method has been previously applied in comparative studies assessing postoperative vaginal length and support after hysterectomy. It provides a practical approximation of vaginal apex descent in the absence of the cervix and allows consistency in comparative analysis between groups.^[17]^

To assess and compare preoperative and postoperative sexual function and symptoms, validated versions of the FSFI, QSES, Pelvic Organ Prolapse Symptom Score (POP-SS), and Female Genital Self-Image Scale (FGSIS) were administered to all patients. Additionally, their partners completed the International Index of Erectile Function (IIEF) questionnaire.

The FSFI questionnaire, consisting of 19 items, was used to assess sexual function over the past 4 weeks. It includes 6 domains: sexual desire (items 1–2), arousal (items 3–6), lubrication (items 7–10), orgasm (items 11–13), sexual satisfaction (items 14–16), and pain (items 17–19). The total score ranges from 2 to 36, with higher scores indicating better sexual function.^[18]^

The QSES is a 7-item questionnaire designed to evaluate changes in the quality of sexual experiences. Scores range from 7 to 49, with higher scores indicating higher quality.^[19]^

The POP-SS questionnaire, comprising 7 items, was used to assess changes in quality of life related to POP symptoms. Each item is scored between 0 and 4, with total scores ranging from 0 to 28. Higher scores indicate more severe symptoms. At the end of the questionnaire, patients were also asked to identify their most bothersome symptom.^[20]^

The FGSIS was used to evaluate patients’ perceptions and beliefs about their genitalia. Scores range from 7 to 28, with higher scores indicating a more positive genital self-image.^[21]^

The IIEF questionnaire was used to assess erectile function in patients’ partners over the past 4 weeks. Scores range from 6 to 30, with higher scores indicating better erectile function.^[22]^

At the 2-year postoperative follow-up, it was confirmed that none of the patients had used medications affecting sexual function, experienced any additional medical issues, undergone other gynecological surgeries, or changed partners.

### 2.1. Surgical procedure

#### 2.1.1. Laparoscopic uterosacral ligament suspension and laparoscopic lateral mesh suspension

A V-shaped mesh was secured to the cervix using a tacker and 2-0 Prolene sutures with the assistance of laparoscopic subperitoneal tunnels. The bilateral transverse mesh arms were pulled until the uterus reached its anatomical position and then anchored to the abdominal wall. Subsequently, the uterosacral ligaments were shortened using sutures.

#### 2.1.2. Bilateral SSLF with VH

After VH, the rectovaginal space was opened up to the vaginal apex. Using blunt dissection, the right pararectal space was accessed, and the ischial spine was palpated as a reference point. The sacrospinous ligament, extending medially from the ischial spine toward the coccyx and the lower sacrum, was identified. The pararectal fascia was perforated, and the space was widened using blunt dissection. With the aid of 2 Breisky-Navratil retractors, the rectum was retracted to the left, exposing the sacrospinous ligament. A nonabsorbable No. 0 Prolene suture was passed through the sacrospinous ligament, approximately 2 to 2.5 cm medial to the ischial spine. The same procedure was performed on the contralateral side. Both ends of the sutures were passed through the vaginal cuff, excess posterior vaginal wall tissue was excised, and the upper one-third of the vaginal mucosa was repaired. After vaginal cuff reconstruction, the sacrospinous sutures were tied near the vaginal cuff apex, suspending the vaginal cuff to the sacrospinous ligaments using 0 Prolene sutures. The same procedure was applied bilaterally.

### 2.2. Statistical analysis

Statistical analyses were conducted using the IBM SPSS Statistics 27 software package (IBM Corporation, Armonk). Frequency tables and descriptive statistics were used to interpret the findings.

For normally distributed measurement values, parametric methods were applied. The “Independent Sample t-test” (t-table value) was used to compare 2 independent groups, while the “Paired Sample t-test” (t-table value) was used to compare 2 dependent groups.

For measurement values that did not follow a normal distribution, nonparametric methods were applied. The “Mann–Whitney U test” (Z-table value) was used to compare 2 independent groups, and the “Wilcoxon test” (Z-table value) was used to compare 2 dependent groups.

Pearson Chi-square (χ^2^) test was used to examine relationships between 2 qualitative variables. ROC curves were utilized to determine the cutoff value of vaginal length based on hysterectomy status.

### 2.3. Power analysis

Using the G*Power 3.1 program, a post hoc power analysis was conducted. The analysis determined that a sample size of 10 patients per group (total of 20 patients) provided 90% power to detect statistically significant differences in primary outcomes (e.g., FSFI scores) with an effect size of *d* = 1.2 and an alpha of 0.05 (two-tailed). This confirms the adequacy of the study design for the planned statistical comparisons.

## 3. Results

A total of 20 female patients, aged between 34 and 49 years, were included in the study. The demographic data of the participants are presented in Table 1.

**Table 1:** Comparison of some quantitative parameters according to groups.

Variable	Group 1 (n = 10)		Group 2 (n = 10)		Statistical analysis* Possibility
	$\bar{X}\pm \;S.D.$	Median [IQR]	$\bar{X}\pm \;S.D.$	Median [IQR]	
Age (yr)	38.70 ± 4.02	39.0 [5.8]	48.10 ± 1.19	48.0 [2.0]	*t* = 7.072 ***P* < .001**
Gravida	3.00 ± 1.05	3.0 [2.0]	3.20 ± 1.55	3.0 [2.3]	*t* = 0.338 *P* = .750
Parity	2.60 ± 1.17	2.0 [1.3]	2.80 ± 1.39	2.0 [2.3]	*Z* = ‐0.203 *P* = .839
Number of previous vaginal births	2.30 ± 1.41	2.0 [1.5]	2.40 ± 1.26	2.0 [1.5]	*t* = 0.166 *P* = .870
Number of previous cesarean births	0.20 ± 0.42	0.0 [0.3]	0.60 ± 0.69	0.5 [1.0]	*Z* = ‐1.446 *P* = .148
Body mass index	24.69 ± 2.12	24.4 [4.2]	26.82 ± 2.01	26.9 [3.4]	*t* = 2.305 ***P* = .033**

Bold values indicate statistical significance (*P* < .05).

S.D. = standard deviation.

* In normally distributed data, “Independent Sample-t” test (t-table value) statistics were used to compare the measurement values of 2 independent groups. “Mann-Whitney U” test (Z-table value) statistics were used to compare the measurement values of 2 independent groups in data that did not have a normal distribution. Group 1 = group with preserved uterus. Group 2 = group with unprotected uterus.

To investigate whether individual demographic or clinical characteristics affected postoperative sexual function outcomes, subgroup comparisons were made between Group 1 (uterus-preserving) and Group 2 (non-uterus-preserving).

Although Group 2 had a significantly higher mean age compared to Group 1 (48.1 ± 1.19 vs 38.7 ± 4.02; *P* < .001), parity and number of previous vaginal or cesarean births were statistically similar between the groups (*P* > .05).

Body mass index (BMI) was slightly higher in Group 2 (26.82 ± 2.01 vs 24.69 ± 2.12; *P* = .033). However, neither age nor BMI was found to significantly correlate with changes in FSFI, QSES, or IIEF scores within either group.

The preoperative and postoperative examination findings of both groups are shown in Table 2. A statistically significant difference was observed between the preoperative and postoperative apical prolapse stages in both groups (*Z* = ‐2.972; *P* = .003) (Table 2). Postoperatively, all patients’ apical prolapse stages regressed to stage 1.

**Table 2:** Comparison of some parameters between groups and within groups.

Variable	Group 1 (n = 10)		Group 2 (n = 10)		Statistical analysis* Possibility
	$\bar{X}\pm \;S.D.$	Median [IQR]	$\bar{X}\pm \;S.D.$	Median [IQR]	
**Apical stage**					
Preoperative	2.80 ± 0.42	3.0 [0.3]	2.80 ± 0.42	3.0 [0.3]	*Z* = 0.000 *P* = 1.000
Postoperative	1.00 ± 0.00	1.0 [0.0]	1.00 ± 0.00	1.0 [0.0]	*Z* = 0.000 *P* = 1.000
Statistical analysis Possibility	*Z* = ‐2.972 ***P* = .003**		*Z* = -2.972 ***P* = .003**		
**Cystocele stage**					
Preoperative	1.90 ± 0.32	2.0 [0.0]	1.70 ± 0.48	2.0 [1.0]	Z = ‐1.090 *P* = .276
Postoperative	1.00 ± 0.00	1.0 [00]	1.00 ± 0.00	1.0 [0.0]	Z = 0.000 *P* = 1.000
Statistical analysis Possibility	*Z* = ‐3.000 ***P* = .003**		*Z* = ‐2.646 ***P* = .008**		
**Rectocele stage**					
Preoperative	1.20 ± 0.42	1.0 [0.3]	1.00 ± 0.00	1.0 [0.0]	*Z* = ‐1.453 *P* = .146
Postoperative	1.00 ± 0.00	1.0 [0.0]	1.00 ± 0.00	1.0 [0.0]	*Z* = 0.000 *P* = 1.000
Statistical analysis Possibility	*Z* = ‐1.414 *P* = .157		*Z* = 0.000 *P* = 1.000		
**Vaginal length**					
Preoperative	14.±1.61	14.5 [3.3]	15.40 ± 0.69	15.5 [1.0]	Z = -1.666 *P* = .096
Postoperative	8.90 ± 0.56	9.0 [0.3]	8.40 ± 0.84	8.0 [1.0]	Z = ‐1.574 *P* = .116
Statistical analysis Possibility	*Z* = ‐2.831 ***P* = .005**		*Z* = ‐2.842 ***P* = .004**		

Bold values indicate statistical significance (*P* < .05).

S.D. = standard deviation.

* “Mann-Whitney U” test (Z-table value) when comparing the measurement values of 2 independent groups in data that does not have a normal distribution; “Wilcoxon” test (Z-table value) statistics were used to compare 2 dependent groups. Group 1 = group with preserved uterus. Group 2 = group with unprotected uterus.

Although there was no statistically significant difference in preoperative and postoperative vaginal length between the groups (*P* > .05), a significant reduction in vaginal length was observed in both groups after surgery (Group 1: *Z* = ‐2.831, *P* = .005; Group 2: *Z* = ‐2.842, *P* = .004) (Table 2).

The questionnaire results assessing sexual function are presented in Tables 3 and 4. While there was a statistically significant difference in postoperative FSFI total scores and FSFI subscale scores between Group 1 and Group 2 (*P* < .05), no significant difference was observed in preoperative scores (*P* > .05). The postoperative scores for sexual desire, arousal, lubrication, orgasm, satisfaction, and pain/discomfort were significantly higher in Group 1 compared to Group 2 (*P* < .05) (Table 3).

**Table 3:** Comparison of FSFI and subscale scores according to groups.

Variable	Group 1 (n = 10)		Group 2 (n = 10)		Statistical analysis* Possibility
	$\bar{X}\pm \;S.D.$	Median [IQR]	$\bar{X}\pm \;S.D.$	Median [IQR]	
**FSFI**					
Preoperative	9.39 ± 1.52	9.3 [3.2]	6.90 ± 5.25	8.3 [9.4]	*t* = ‐1.441 *P* = .179
Postoperative	31.59 ± 0.71	31.7 [0.8]	22.06 ± 1.16	22.2 [2.2]	*t* = ‐22.011 ***P* < .001**
Statistical analysis Possibility	*t* = ‐36.098 ***P* < .001**		*t* = ‐9.862 ***P* < .001**		
**Desire**					
Preoperative	1.56 ± 0.42	1.5 [0.6]	1.44 ± 0.42	1.2 [0.6]	Z = ‐0.781 *P* = .435
Postoperative	4.32 ± 0.55	4.5 [1.2]	3.30 ± 0.42	3.6 [0.6]	Z = ‐3.283 ***P* = .001**
Statistical analysis Possibility	*Z* = ‐2.827 ***P* = .005**		*Z* = ‐2.836 ***P* = .005**		
**Arousal**					
Preoperative	1.98 ± 0.20	2.0 [0.3]	1.23 ± 1.15	1.4 [2.4]	Z = ‐0.739 *P* = .460
Postoperative	5.01 ± 0.28	4.8 [0.6]	3.60 ± 0.66	3.3 [0.7]	Z = ‐3.459 ***P* < .001**
Statistical analysis Possibility	*Z* = ‐2.831 ***P* = .005**		*Z* = ‐2.816 ***P* = .005**		
**Lubrication**					
Preoperative	1.29 ± 0.20	1.2 [0.1]	1.11 ± 1.21	1.2 [1.7]	*Z* = ‐1.200 *P* = .230
Postoperative	5.94 ± 0.12	6.0 [0.1]	3.84 ± 0.28	3.9 [0.3]	*Z* = ‐3.954 ***P* < .001**
Statistical analysis Possibility	*Z* = ‐2.913 ***P* = .004**		*Z* = ‐2.810 ***P* = .005**		
**Orgasm**					
Preoperative	1.44 ± 0.39	1.2 [0.4]	0.96 ± 0.91	1.2 [1.7]	*Z* = ‐1.199 *P* = .230
Postoperative	5.28 ± 0.17	5.2 [0.1]	3.68 ± 0.25	3.6 [0.4]	*Z* = ‐3.969 ***P* < .001**
Statistical analysis Possibility	*Z* = ‐2.831 ***P* = .005**		*Z* = ‐2.814 ***P* = .005**		
**Satisfaction**					
Preoperative	1.44 ± 0.27	1.4 [0.4]	1.16 ± 1.06	1.4 [2.1]	*Z* = ‐0.116 *P* = .907
Postoperative	5.04 ± 0.39	4.8 [0.4]	3.64 ± 0.12	3.6 [0.0]	*Z* = ‐4.028 ***P* < .001**
Statistical analysis Possibility	*Z* = ‐2.820 ***P* = .005**		*Z* = ‐2.829 ***P* = .005**		
**Pain**					
Preoperative	1.68 ± 0.81	1.2 [0.8]	1.00 ± 0.92	1.2 [1.7]	Z = ‐1.103 *P* = .270
Postoperative	6.00 ± 0.00	6.0 [0.0]	4.00 ± 0.26	4.0 [0.0]	*Z* = ‐4.104 ***P* < .001**
Statistical analysis Possibility	*Z* = ‐2.873 ***P* = .004**		*Z* = ‐2.809 ***P* = .005**		

Bold values indicate statistical significance (*P* < .05).

FSFI = Female Sexual Function Index, S.D. = standard deviation.

* “Mann-Whitney U” test (Z-table value) when comparing the measurement values of 2 independent groups in data that does not have a normal distribution; “Wilcoxon” test (Z-table value) statistics were used to compare 2 dependent groups. Group 1 = group with preserved uterus. Group 2 = group with unprotected uterus.

**Table 4:** Comparison of QSES, POP-SS, FGSIS, and FGSIS subdimensions according to groups.

Variable	Group 1 (n = 10)		Group 2 (n = 10)		Statistical analysis* Possibility
	$\bar{X}\pm \;S.D.$	Median [IQR]	$\bar{X}\pm \;S.D.$	Median [IQR]	
**QSES**					
Preoperative	10.50 ± 1.65	10.5 [2.3]	10.80 ± 3.04	10.0 [3.8]	*t* = 0.274 *P* = .787
Postoperative	42.60 ± 1.95	42.5 [3.3]	27.50 ± 2.50	28.0 [1.5]	Z = ‐3.817 ***P* < .001**
Statistical analysis Possibility	*t* = ‐42.684 ***P* < .001**		*Z* = ‐2.809 ***P* = .005**		
**POP-SS**					
Preoperative	23.50 ± 2.59	24.0 [3.0]	16.70 ± 2.16	17.0 [3.5]	*Z* = ‐3.577 ***P* < .001**
Postoperative	4.40 ± 1.89	4.5 [3.0]	1.10 ± 1.91	0.0 [2.5]	Z = ‐3.024 ***P* = .002**
Statistical analysis Possibility	*Z* = ‐2.812 ***P* = .005**		*Z* = ‐2.810 ***P* = .005**		
**FGSIS**					
Preoperative	9.90 ± 0.73	10.0 [1.3]	11.20 ± 1.93	11.0 [2.3]	*Z* = ‐1.925 *P* = .054
Postoperative	25.60 ± 0.96	26.0 [0.3]	20.90 ± 0.88	21.0 [1.0]	*Z* = ‐3.930 ***P* < .001**
Statistical analysis Possibility	*Z* = ‐2.825 ***P* = .005**		*Z* = ‐2.825 ***P* = .005**		
**Orgasmic function**					
Preoperative	8.70 ± 0.48	9.0 [1.0]	5.30 ± 4.57	8.5 [9.0]	Z = ‐1.378 *P* = .168
Postoperative	10.00 ± 0.00	10.0 [0.0]	9.10 ± 0.58	9.0 [0.3]	Z = ‐3.508 ***P* < .001**
Statistical analysis Possibility	*Z* = ‐2.919 ***P* = .004**		*Z* = ‐2.271 ***P* = .023**		
**Desire**					
Preoperative	6.80 ± 1.03	6.0 [2.0]	5.80 ± 1.99	6.0 [4.0]	Z = ‐1.159 *P* = .246
Postoperative	10.00 ± 0.00	10.0 [0.0]	7.70 ± 0.67	8.0 [0.3]	Z = ‐4.192 ***P* < .001**
Statistical analysis Possibility	*Z* = ‐2.889 ***P* = .004**		*Z* = ‐2.414 ***P* = .016**		
**Sexual satisfaction**					
Preoperative	4.40 ± 0.84	4.0 [1.0]	3.30 ± 2.86	5.0 [6.0]	*Z* = ‐0.078 *P* = .938
Postoperative	13.40 ± 1.58	13.5 [3.0]	9.90 ± 0.74	10.0 [1.3]	*Z* = ‐3.764 ***P* < .001**
Statistical analysis Possibility	*Z* = ‐2.814 ***P* = .005**		*Z* = ‐2.820 ***P* = .005**		
**Overall satisfaction**					
Preoperative	3.10 ± 0.87	3.0 [2.0]	2.80 ± 1.03	2.0 [2.0]	*Z* = ‐0.741 *P* = .459
Postoperative	10.00 ± 0.00	10.0 [0.0]	6.80 ± 1.03	6.0 [2.0]	*Z* = ‐4.119 ***P* < .001**
Statistical analysis Possibility	*Z* = ‐2.836 ***P* = .005**		*Z* = ‐2.836 ***P* = .005**		

Bold values indicate statistical significance (*P* < .05).

FGSIS = Female Genital Self-Image Scale, POP-SS = Pelvic Organ Prolapse Symptom Score, QSES =Quality of Sexual Experience Scale, S.D. = standard deviation.

* “Mann-Whitney U” test (Z-table value) when comparing the measurement values of 2 independent groups in data that does not have a normal distribution; “Wilcoxon” test (Z-table value) statistics were used to compare 2 dependent groups. Group 1 = group with preserved uterus. Group 2 = group with unprotected uterus.

When preoperative and postoperative scores were compared within each group, postoperative QSES, FGSIS, and FGSIS subscale scores were significantly higher than preoperative values, while POP-SS scores were significantly lower (*P* < .05).

The postoperative erectile function scores of partners in Group 1 were significantly higher than those in Group 2 (Table 4).

Overall, when the questionnaire scores of both groups were compared, Group 1 patients had statistically significantly higher scores than Group 2 (*P* < .05).

At the 2-year postoperative follow-up, all patients were evaluated for recurrence, ongoing symptoms, and sustained improvements in sexual and genital perception scores. None of the patients in either group experienced recurrence of apical prolapse, and all maintained a stage 1 or lower prolapse based on POP-Q assessment.

Additionally, no further gynecological procedures, complications, or partner changes were reported during this period.

The improvements observed in FSFI, QSES, and FGSIS scores were sustained throughout the 2-year follow-up. This indicates long-term durability of the sexual function benefits, particularly in Group 1. The POP-SS scores also remained stable without significant symptom recurrence.

## 4. Discussion

This study investigated the impact of surgical techniques used for the treatment of isolated apical prolapse on female and male sexual functions, considering the presence of the uterus and cervix. The findings indicate that uterus-preserving surgical techniques, such as LLMS and LUSLS, resulted in better outcomes in terms of symptom relief, sexual function, and partner satisfaction compared to VH with bilateral SSLF. It is suggested that preserving the vascular and neural structures around the cervical ring during prolapse surgery may contribute to improved sexual function and partner satisfaction.

The uterus and cervix play a crucial role in maintaining pelvic floor structure and function. In particular, the continuity of the cervical ring is essential for sexual function.^[23]^ Masters et al reported that procedures such as subtotal hysterectomy help maintain normal sexual function by preserving the cervical ring and its surrounding nerves.^[24]^ These findings highlight the erogenous nature of the cervix and its neural structures. The weakening of pelvic floor ligaments following hysterectomy, along with reduced blood supply to the cardinal and uterosacral ligaments, increases the risk of prolapse, while the loss of neural stimulation in the cervical region may lead to decreased sexual satisfaction.

Costantini et al demonstrated that among patients undergoing surgery for POP, those who underwent sacrocolpopexy with uterine preservation showed significantly better sexual function compared to those who underwent hysterectomy with sacrocolpopexy.^[25]^ Veitrubin et al., in a large-scale review, emphasized that LLMS preserves sexual function.^[15]^ On the other hand, Schulten et al reported no significant differences in quality of life and sexual function in a 5-year follow-up of patients who underwent sacrospinous hysteropexy and uterosacral ligament suspension alongside VH.^[26]^

These findings suggest that there is no clear consensus on the most beneficial surgical technique for POP treatment. However, preserving the uterus and cervical ring appears to be a key factor in maintaining sexual function. In our study, Group 1 underwent LLMS and LUSLS, while Group 2 underwent VH and SSF. Both groups showed regression of prolapse to stage 1 and improvements in sexual function. However, the improvement was statistically more significant in Group 1 than in Group 2. The preservation of the uterus and cervical ring could have contributed to the observed difference. In Group 1, where the uterus was preserved, the maintenance of the cervical ring and vaginal fornices, as well as the preservation of erogenous zones, may have played a role in the observed improvement in sexual function. In contrast, in Group 2, where hysterectomy was performed, the decline in sexual function is thought to be related to nerve damage in the vaginal tissues, the loss of uterine contractions, and a greater reduction in vaginal length.

Shah et al used the FSFI questionnaire to assess sexual function after vaginal POP repair and found significant improvements in women.^[27]^ Similarly, Azar et al found statistically significant improvements in sexual desire, arousal, lubrication, orgasm, and satisfaction following POP surgery as assessed by FSFI.^[28]^ In line with these findings, significant improvements were observed in FSFI, QSES, and FGSIS scores in both groups in this study. Although minimal important difference thresholds for all subscales are not universally defined, the magnitude of the observed differences (especially in postoperative FSFI and FGSIS scores) indicates clinically meaningful improvement, particularly in orgasmic function and pain domains.

Several studies have also examined partner satisfaction in relation to hysterectomy. Lalos et al reported that most men felt their partners’ preoperative POP symptoms had negatively affected their own sexual lives, and that they experienced improvements after the surgery.^[29]^ However, Newman et al found that some men perceived their partners as less desirable after hysterectomy and even refused sexual intercourse.^[30]^ In our study, based on the IIEF questionnaire, partner satisfaction and erectile function scores were significantly higher in Group 1 compared to Group 2. This finding suggests that the presence of the uterus also has a substantial effect on male sexuality.

The vaginal relaxation and the weight of prolapsed uterus and tissues in POP negatively impact both functional and sexual well-being. In this study, postoperative vaginal relaxation improved in both groups, and total vaginal length was reduced. However, the reduction in vaginal length was greater in Group 2 (VH + SSF) compared to Group 1. Despite this, sexual function improved significantly in both groups, with a more pronounced improvement in Group 1 (*P* < .05). The literature includes studies showing that, in cases where hysterectomy is performed for indications other than POP and where vaginal relaxation is absent, the shortening of vaginal length does not have a clear effect on sexual function.^[31]^ If the vagina is shorter than normal, it may cause pain, especially in deep penetration, especially if sufficient stretching cannot be provided during sexual intercourse. Because the vagina expands and lengthens during sexual stimulation and contributes to the woman’s orgasm. A short vagina can reduce sensitivity and pleasure in some women, making it difficult to reach orgasm with penetration.^[32]^

Although both surgical groups exhibited a statistically significant reduction in vaginal length postoperatively, the reduction was more pronounced in Group 2 (VH with bilateral SSLF). This can be attributed to the removal of the uterus and cervix, which eliminates the natural apical support and contributes to foreshortening of the vaginal canal. Furthermore, the fixation of the vaginal cuff directly to the sacrospinous ligaments may alter the axis and curvature of the upper vagina, leading to decreased vaginal elasticity and potential apical shortening. In contrast, uterus-preserving techniques (LLMS and LUSLS) maintain the cervical ring and upper vaginal fornices, preserving the anatomical length and contour of the vaginal vault.^[33]^

These findings may indicate that preserving the uterus and cervical ring is associated with better psychological well-being and sexual function, possibly due to the retention of erogenous zones in the cervix and surrounding structures.

In POP surgery, suspension procedures such as SSF are frequently performed alongside VH. While unilateral sacrospinous ligament fixation may lead to vaginal axis deviation and sexual dysfunction, bilateral SSF has been shown to improve sexual function. Maintaining vaginal anatomical symmetry is essential for sexual function.^[34]^ Şentürk et al stated that bilateral SSF helps restore the vaginal axis to its original anatomical position, which enhances sexual function.^[35]^ Nicolau-Toulouse et al reported that the vaginal apex-to-ischial spine distance measured by MRI in patients undergoing bilateral SSF was similar to that in nulliparous women.^[36]^

In recent years, robot-assisted sacrocolpopexy (RASC) has gained prominence in minimally invasive apical prolapse surgery. RASC offers similar anatomical and functional results when compared to laparoscopic methods; however, it has been associated with advantages such as reduced blood loss, lower intraoperative complication rates.^[37]^

In randomized controlled trials, the operative time was longer than laparoscopic sacrocolpopexy; however, there was no difference in clinical outcomes during patient recovery.^[38]^ In addition, single-port robotic techniques offer cosmetic advantage and potentially lower postoperative pain profile by allowing operation through a single incision of only 2.5 to 3.5 cm.^[39]^

In our study, uterus-sparing techniques (LLMS and LUSLS) achieved favorable results in terms of both economical operation time and significant improvement in sexual function. On the other hand, RASC has logistical limitations such as accessibility, learning curve, device cost.^[38]^

One of the strengths of this study is that there was no statistically significant difference between the groups in terms of gravida, parity, and mode of delivery, allowing for a more reliable comparison. Additionally, all surgeries were performed by an experienced surgeon under standardized conditions, minimizing inconsistencies between the groups. The exclusive focus on isolated apical prolapse, without additional conditions such as cystocele that could affect sexual satisfaction, was another important factor ensuring the accuracy of evaluations.

## 5. Limitations and prospects

Although the age difference between the groups in our study is notable, this variable alone does not invalidate the primary outcomes of the study. The study groups consisted of female patients with comparable baseline sexual lives and sexual function profiles. Therefore, the observed change in sexual satisfaction after surgery is more likely to reflect the effect of the surgical intervention rather than age-related differences.

Nevertheless, we acknowledge that both age and BMI may act as potential confounding factors influencing sexual function outcomes. Accordingly, these variables should be considered in the interpretation of the results and represent a methodological limitation of the study. Future studies should incorporate multivariable adjustment models or covariance analyses to control for these confounders and to strengthen causal inference.

In addition, the small sample size (n = 20) limits the generalizability of the findings and reduces the statistical power of the analyses. Conducting the study with a larger patient population would improve the reliability and external validity of the results.

Large-scale studies evaluating different surgical techniques and combined procedures may provide important contributions to the literature. In the future, hybrid techniques such as robotic-assisted sacrocolpopexy, single-port laparoscopic, or robotic abdominal approaches may be increasingly used in clinical practice, offering both patient convenience and improved surgeon ergonomics as well as cost optimization. When evaluated together with the results of our study, these developments may provide a basis for investigating alternative surgical approaches.

## 6. Conclusion

Surgical interventions for POP are known to alleviate prolapse-related symptoms and may also contribute to improvements in sexual function. In this study, patients who underwent uterus- and cervix-preserving procedures experienced greater improvements in sexual function compared to those who underwent hysterectomy-based approaches. While the exact mechanism remains unclear, the preservation of the uterus and cervical ring, along with associated neurovascular structures, may play a role in maintaining sexual well-being. These findings suggest that, when feasible, preserving the cervical ring might be beneficial and should be considered in the surgical planning process.

## Author contributions

**Conceptualization:** Elif Ucar, Murat Yassa.

**Data curation:** Elif Ucar, Ozan Dogan.

**Formal analysis:** Ozan Dogan.

**Funding acquisition:** Elif Ucar, Ozan Dogan.

**Investigation:** Murat Yassa.

**Methodology:** Elif Ucar, Murat Yassa.

**Resources:** Elif Ucar, Ozan Dogan.

**Software:** Elif Ucar, Erdem Gürkan.

**Supervision:** Ozan Dogan, Murat Yassa.

**Validation:** Erdem Gürkan.

**Visualization:** Erdem Gürkan.

**Writing – original draft:** Elif Ucar, Erdem Gürkan.

**Writing – review & editing:** Murat Yassa.

## References

[1] Wu JM Vaughan CP Goode PS. Prevalence and trends of symptomatic pelvic floor disorders in U.S. women. Obstet Gynecol. 2014;123:141–8. 24463674 10.1097/AOG.0000000000000057 PMC3970401

[2] Wu JM Hundley AF Fulton RG Myers ER. Forecasting the prevalence of pelvic floor disorders in U.S. Women: 2010 to 2050. Obstet Gynecol. 2009;114:1278–83. 19935030 10.1097/AOG.0b013e3181c2ce96

[3] Kuo CH Martingano DJ Mikes BA. Pelvic organ prolapse. In: StatPearls. StatPearls Publishing; 2025. 33085376

[4] Zumrutbas AE. Understanding pelvic organ prolapse: a comprehensive review of etiology. Soc Int Urol J. 2025;6:6.

[5] Sartori DVB Kawano PR Yamamoto HA Guerra R Pajolli PR Amaro JL. Pelvic floor muscle strength is correlated with sexual function. Investig Clin Urol. 2021;62:79–84. 10.4111/icu.20190248 PMC7801170 33258326

[6] Martins FE. Pelvic organ prolapse and sexual dysfunction. Soc Int Urol J. 2025;6:19.

[7] Wihersaari O Karjalainen P Tolppanen AM Mattsson N Nieminen K Jalkanen J. Sexual activity and dyspareunia after pelvic organ prolapse surgery: a 5-year nationwide follow-up study. Eur Urol Open Sci. 2022;45:81–9. 36353662 10.1016/j.euros.2022.09.014 PMC9637561

[8] Antosh DD Kim-Fine S Meriwether KV. Changes in sexual activity and function after pelvic organ prolapse surgery: a systematic review. Obstet Gynecol. 2020;136:922–31. 33030874 10.1097/AOG.0000000000004125

[9] Ferrari A Bellè N Giannini A Simoncini T Vainieri M. Determinants of women’s preferences for surgical versus conservative management for pelvic organ prolapse: a survey-based study from Italy. BMJ Open. 2024;14:e084034. 10.1136/bmjopen-2024-084034 PMC11284879 39053952

[10] Brennand EA Scime NV Huang B. Hysterectomy versus uterine preservation for pelvic organ prolapse surgery: a prospective cohort study. Am J Obstet Gynecol. 2025;232:461.e1–20. 10.1016/j.ajog.2024.10.021 39428029

[11] Carlin GL Hummel Jiménez J Lange S. Impact on sexual function and wish for subsequent pregnancy after uterus-preserving prolapse surgery in premenopausal women. J Clin Med. 2024;13:4105. 39064144 10.3390/jcm13144105 PMC11277568

[12] Ferhi M Marwen N Abdeljabbar A Mannai J. To preserve or not to preserve: a prospective cohort study on the role of the cervix in post-hysterectomy sexual functioning. Cureus. 2024;16:e68876. 39376845 10.7759/cureus.68876 PMC11457895

[13] Barber MD Maher C. Apical prolapse. Int Urogynecol J. 2013;24:1815–33. 24142057 10.1007/s00192-013-2172-1

[14] Dubuisson JB Yaron M Wenger JM Jacob S. Treatment of genital prolapse by laparoscopic lateral suspension using mesh: a series of 73 patients. J Minim Invasive Gynecol. 2008;15:49–55. 18262144 10.1016/j.jmig.2007.11.003

[15] Veit-Rubin N Dubuisson J Gayet-Ageron A Lange S Eperon I Dubuisson J. Patient satisfaction after laparoscopic lateral suspension with mesh for pelvic organ prolapse: outcome report of a continuous series of 417 patients. Int Urogynecol J. 2017;28:1685–93. 28417156 10.1007/s00192-017-3327-2

[16] Dancz CE Werth L Sun V Lee S Walker D Özel B. Comparison of the POP-Q examination, transvaginal ultrasound, and direct anatomic measurement of cervical length. Int Urogynecol J. 2014;25:457–64. 24170226 10.1007/s00192-013-2255-z

[17] Persu C Chapple CR Cauni V Gutue S Geavlete P. Pelvic organ prolapse quantification system (POP-Q) – a new era in pelvic prolapse staging. J Med Life. 2011;4:75–81. 21505577 PMC3056425

[18] Aygin D Eti Aslan F. Kadin Cinsel İşlev Ölçeği’nin Türkçeye Uyarlamasi. Turkiye Klinikleri J Med Sci. 2005;25:393–9.

[19] Yassa M Sargin MA Dereli N. The validity and reliability of the Turkish version of the quality of sexual experience scale. Anatolian J Psychiatry. 2020;21:48–55.

[20] Özengin N Kaya S Orhan C. Turkish adaptation of the pelvic organ prolapse symptom score and its validity and reliability. Int Urogynecol J. 2017;28:1217–22. 28062904 10.1007/s00192-016-3251-x

[21] Ellibes Kaya A Yassa M Dogan O Basbug A Pulatoglu C Caliskan E. The female genital self-image scale (FGSIS): cross-cultural adaptation and validation of psychometric properties within a Turkish population. Int Urogynecol J. 2019;30:89–99. 29961112 10.1007/s00192-018-3688-1

[22] Bayraktar Z. The reliability of the Turkish version of the international index of erectile function (IIEF): literature review. New J Urol. 2017;12:63–70.

[23] Petros P. Influence of hysterectomy on pelvic-floor dysfunction. Lancet. 2000;356:1275. 10.1016/S0140-6736(05)73879-4 11072974

[24] Masters W Johnson V. Human Sexual Response. Little, Brown, Co; 1966

[25] Costantini E Porena M Lazzeri M Mearini L Bini V Zucchi A. Changes in female sexual function after pelvic organ prolapse repair: role of hysterectomy. Int Urogynecol J. 2013;24:1481–7. 23361855 10.1007/s00192-012-2041-3

[26] Schulten SFM Detollenaere RJ Stekelenburg J IntHout J Kluivers KB Van Eijndhoven HWF. Sacrospinous hysteropexy versus vaginal hysterectomy with uterosacral ligament suspension in women with uterine prolapse stage 2 or higher: observational follow-up of a multicentre randomised trial. BMJ. 2019;366:l5149. 31506252 10.1136/bmj.l5149 PMC6734519

[27] Shah SM Bukkapatnam R Rodriguez LV. Impact of vaginal surgery for stress urinary incontinence on female sexual function: is the use of polypropylene mesh detrimental? Urology. 2005;65:270–4. 15708036 10.1016/j.urology.2004.08.058

[28] Azar M Noohi S Radfar S Radfar MH. Sexual function in women after surgery for pelvic organ prolapse. Int Urogynecol J. 2007;19:53–7. 10.1007/s00192-007-0399-4 17571198

[29] Lalos A Lalos O. The partner’s view about hysterectomy. J Psychosom Obstet Gynecol. 1996;17:119–24. 10.3109/01674829609025671 8819022

[30] Newman G Newman L. Coping with the stress of hysterectomy. J Sex Educ Ther. 1985;11:65–8.

[31] Dedden SJ Werner MA Steinweg J. Hysterectomy and sexual function: a systematic review and meta-analysis. J Sex Med. 2023;20:447–66. 36857309 10.1093/jsxmed/qdac051

[32] Abdelmonem AM. Vaginal length and incidence of dyspareunia after total abdominal versus vaginal hysterectomy. Eur J Obstet Gynecol Reprod Biol. 2010;151:190–2. 20427116 10.1016/j.ejogrb.2010.03.031

[33] Medina CA Croce C Candiotti K Takacs P. Comparison of vaginal length after iliococcygeus fixation and sacrospinous ligament fixation. Int J Gynaecol Obstet. 2008;100:267–70. 18022624 10.1016/j.ijgo.2007.09.018

[34] Wang K Shi L Huang Z Xu Y. Bilateral sacrospinous hysteropexy versus bilateral sacrospinous ligament fixation with vaginal hysterectomy for apical uterovaginal prolapse. Int Neurourol J. 2022;26:239–47. 36203256 10.5213/inj.2244076.038 PMC9537431

[35] Şentürk MB Güraslan H Çakmak Y Ekin M. Bilateral sacrospinous fixation without hysterectomy: 18-month follow-up. J Turk Ger Gynecol Assoc. 2015;16:102–6. 26097393 10.5152/jtgga.2015.15220 PMC4456967

[36] Nicolau-Toulouse V Tiwari P Lee T Cundiff GW Geoffrion R. Does bilateral sacrospinous fixation with synthetic mesh recreate nulliparous pelvic anatomy? An MRI evaluation. Female Pelvic Med Reconstr Surg. 2014;20:222–7. 24978089 10.1097/SPV.0000000000000066

[37] Evangelopoulos N Nessi A Achtari C. Minimally invasive sacrocolpopexy: efficiency of robotic assistance compared to standard laparoscopy. J Robot Surg. 2024;18:72. 38340232 10.1007/s11701-023-01799-1 PMC10858822

[38] Callewaert G Bosteels J Housmans S. Laparoscopic versus robotic-assisted sacrocolpopexy for pelvic organ prolapse: a systematic review. Gynecol Surg. 2016;13:115–23. 27226787 10.1007/s10397-016-0930-z PMC4854942

[39] Nam G Lee SR Roh AM. Single-incision vs. multiport robotic sacrocolpopexy: 126 consecutive cases at a single institution. J Clin Med. 2021;10:4457. 34640475 10.3390/jcm10194457 PMC8509716

